# Academic Self-Efficacy, Social Support, and Professional Identity Among Preservice Special Education Teachers in China

**DOI:** 10.3389/fpsyg.2020.00374

**Published:** 2020-03-13

**Authors:** Xiaomeng Chen, Jingxun Zhong, Manli Luo, Minghui Lu

**Affiliations:** ^1^Special Education Department, School of Education, South China Normal University, Guangzhou, China; ^2^Institute of Educational Administration, School of Education, South China Normal University, Guangzhou, China; ^3^Futian Zhuxiang School, Shenzhen, China; ^4^Special Education Department, School of Education, Guangzhou University, Guangzhou, China

**Keywords:** social support, mediating effect, academic self-efficacy, professional identity, preservice special education teachers

## Abstract

The relationship among the social support, professional identity, and academic self-efficacy (ASE) of Chinese preservice special education teachers are explored by measuring the perceived social support, professional identity, and ASE of 302 undergraduate students. Results of the multiple regression are as follows. (1) A significant positive correlation exists among ASE, social support, and professional identity. When preservice special education teachers perceive high social support, they have a high sense of professional identity and high ASE. (2) Professional identity exerts a full mediation effect on the relationship between social support and ASE. In particular, social support positively influences ASE via professional identity. The results are discussed at the end of this paper and recommendations for improving the ASE of preservice special education teachers are presented.

## Introduction

Teacher education has long played an intuitive and important role in special education. At present, the preservice education of special education teachers in China is carried out mainly by normal colleges specializing in special education teacher education and normal universities offering special education courses (Wang and Mu, 2014). Currently, more than 50 such specialized educational institutions can be found throughout the country, most of which have been established since 2000 (Wang and Mu, 2014). Meanwhile, in the United States, a variety of incubator programs, such as four-year teacher preservice programs and alternative routes, among others, have been established (Sindelar et al., 2010). Preservice special education teachers in China have not undertaken any special education teaching work and thus are referred to as student teachers in this paper. Although special education teacher education has made remarkable strides in China, few studies have focused on the psychological state of these student teachers. From the limited research, the learning status of preservice special education teachers in China may not be ideal, especially their learning motivation is weak. Chen and Yang (2018) surveyed 538 preservice special education teachers and found that 58.9% of them did not choose this major voluntarily and that their lack of in-depth knowledge of this major led them to think they were not suitable for this specialty during their study. Moreover, the lack of social support during the learning process of student teachers may also affect their enthusiasm for learning (Zhao and Zhang, 2017).

Academic self-efficacy (ASE) refers to a person’s belief that he or she has the ability to complete the academic tasks prescribed by the school. It plays an important role among preservice special education teachers because it generally determines the learning motivation and academic achievement of students (Richardson et al., 2012; Chen and Yang, 2018). At present, numerous college students in China are involuntarily majoring in special education, which lowers their professional identity (Chen and Yang, 2018). Professional identity is defined as a person’s sense of belonging to and recognition of the profession (Tickle, 1999), which has an important influence on individual career learning and engagement. Furthermore, the public remains unfamiliar with the special education profession and has biased views of special education teachers (Lu et al., 2018). Taken together, these factors may affect the ASE of preservice special education teachers.

Although the relationship among ASE, social support, and professional identity has been identified by several authors (Day et al., 2006; Tschannen-Moran and Hoy, 2007; Adler-Constantinescu et al., 2013; Chen and Yang, 2018), to date, little research has been conducted into the role of professional identity in the relationship between social support and ASE among preservice special education teachers. Moreover, in contrast with numerous western countries, knowledge regarding preservice special education teacher programs in China remains limited. Thus, the objective of the current study is to investigate the relationship among the ASE, social support, and professional identity of Chinese preservice special education teachers. Therefore, improving the ASE and related factors, such as social support and professional identity, of preservice special education teachers is crucial.

## Literature Review

### Academic Self-Efficacy

Bandura (1997) defined self-efficacy as “the belief in one’s capabilities to organize and execute courses of action required to produce given attainments.” In accordance with social cognitive theory, self-efficacy affects many areas of people’s lives, including their goals, decisions, effort levels, thinking patterns, and the perseverance level they maintain in the face of challenges (Bandura, 1991). Zimmerman (2000) pointed out that people’s self-efficacy varies depending on domains. In the academic context, self-efficacy is generally identified as ASE (Elias and MacDonald, 2007). ASE refers to a person’s belief in organizing, managing, and executing actions to achieve his/her desired academic performance (Zimmerman et al., 1992). Students with a high ASE level are confident in their ability to meet academic requirements, plan and organize their education, and avoid distractions (Bandura, 1997). ASE is positively correlated with academic performance. When students have a high sense of learning efficacy, they become more confident that they can overcome difficulties, work hard, and ultimately improve their academic performance (Richardson et al., 2012; Komarraju and Nadler, 2013). Similarly, the results of the meta-analysis of Richardson et al. (2012) indicate that ASE beliefs can explain 9% of the variance in the overall grade point average of college students. Furthermore, Byer (2002) found that developing a positive ASE is important among graduate students because such ASE will lead them to believe that they can achieve their curriculum and degree goals. Students’ belief in the mastery of academic activities can determine their academic motivation and achievement (Bandura, 1993).

In recent years, Chinese researchers have investigated the learning situation of preservice special education teachers and found that their ASE is only at a medium level (it is in the middle level on the score of Likert Scale), which may negatively affect the learning initiative of students (Guan et al., 2011; Chen and Yang, 2018). However, a study by Gur (2012) of Israeli preservice special education teachers found that student teachers had above medium ASE, in particular, each item of self-efficacy was measured according to a scale of 1–5, whereby 1 was the lowest score, 5 the highest score, and 3 at the intermediate level, the participants scored close to 4, exceeding the intermediate level. Although numerous studies have described the relationship between ASE and academic achievement, the relationship between ASE and other variables, such as professional identity, social support, and demographic background, has seldom been researched. To improve the understanding of the relationship between ASE and these variables in the future, other related variables should also be studied.

### Social Support and ASE

The development of the ASE of preservice special education teachers may require social support (Lombardo-Graves, 2014). Social support refers to physical or psychological assistance received by people through social connections. Social support can reduce psychological pressure, alleviate tension, and improve people’s ability to adapt to society (Cobb, 1976). The higher the perceived level of support from social networks, the more people rethink their current difficult situations, the higher their confidence, and the lower the threat of emotional responses like anxiety and fear, thereby resulting in appropriate coping strategies (Sippel et al., 2015). Adler-Constantinescu et al. (2013) found a positive correlation between self-efficacy and perceived social support among adolescents. In particular, the more support received from parents and friends during early adolescence, the higher the self-efficacy level during late adolescence. Social support is an important factor that determines teachers’ self-efficacy and mental health (Wallace et al., 2001; Shen, 2009; Nabavi et al., 2017). Tschannen-Moran and Hoy (2007) determined that compared with that of experienced teachers, the perceived support of young, inexperienced teachers from others exerts a greater impact on their self-efficacy. From this perspective, social support is highly significant in fostering Chinese special education student teachers. Lu et al. (2018) surveyed 1,027 special education teachers in China and found that although their social support directly affects their self-efficacy, it also exerts an indirect effect on their self-efficacy through work engagement. However, the aforementioned studies have been aimed at in-service teachers, and whether such a relationship exists among preservice teachers remains unknown.

### Relationship Among Professional Identity, Social Support, and ASE

In addition to social support, professional identity may also affect the ASE of preservice teachers. Professional identity refers to an individual’s identity and sense of belonging to a profession (Tickle, 1999). Professional identity considerably influences teachers’ professional development, curriculum and teaching, and loyalty to the teaching profession (Korthagen, 2004). It can alleviate job burnout, improve job satisfaction (Beijaard et al., 2000; Hong, 2010), and predict the degree of involvement in professional preparation among preservice teachers (Zhang Y. et al., 2016). Preservice special education teachers are not only ordinary college students but are also preservice teachers with a teacher career orientation, and thus, their university learning and training are closely related to their future in the teaching profession. Their professional identity may significantly impact their university learning experience.

Studies have shown that identity is related to and can predict ASE (Kerpelman et al., 2008; Komarraju and Dial, 2014; Chen and Yang, 2018). For example, when people believe in their identity and their actions are congruent, they generally tend to take on challenging tasks and experience the meaning of those tasks (Oyserman and Destin, 2010). Moore and Hofman (1988) showed that the professional identity of teachers affects their professional development and sense of efficacy because teachers with a positive self-perception of his/her professional identity will ignore unpleasant working conditions. Chen and Yang (2018) also found a positive correlation between professional identity and ASE; that is, the ASE of special education teachers increases with the improvement of professional identity. Perceived high levels of professional identity can promote motivation to learn and interest in learning, diligent study habits, and mastery of special education knowledge and skills among preservice special education teachers, all of which help them to attain academic success, experience a sense of achievement and pleasure, and maintain a high level of ASE.

Although professional identity is important, its level is generally low in China (Guan et al., 2011). As mentioned above, Chinese society generally perceives special education teachers as having a lower social status than regular teachers, which undermines the professional identity of preservice special education teachers. However, like other majors in China, such as preschool teachers, their professional identity is above average (Hu and Sang, 2013). Therefore, the improvement of the professional identity of preservice special education teachers necessitates society’s care and support. Numerous studies have focused on the relationship between social support and professional identity in China (Wei and Zhang, 2009; Sun and Lei, 2016; Zhang and Wang, 2018). For example, Sun and Lei (2016) found that after entering university, preservice teachers lacked family support, received more direct support from their classmates and teachers, and the more care they felt, the more they identified with special education majors. Johnson and Ridley (2004) determined that providing sufficient support to preservice teachers, including initial instructions to novice teachers, learning the school culture, and communicating classroom plans with experienced teachers, can reduce the difficulty of the transition from student to teacher. Similarly, studies on novice teachers have shown that support and positive feedback from supervisors, assistants, and parents are critical to the success and well-being of these teachers (Avalos and Aylwin, 2007; Oplatka and Eizenberg, 2007). Therefore, providing sufficient social support to preservice teachers may help them actively identify with the teaching profession, which may enhance their ASE.

## Theory Development

In summary, previous studies focused on the relationship between social support and ASE and the relationship between professional identity and ASE, but integrated research on the relationship between the three has been limited or even non-existent. Although social support is associated with ASE, far less is known about the mechanisms underlying this relationship. In particular, professional identity may play an important mediating role between the two; that is, social support may affect the professional identity of preservice teachers in special education and the promotion of professional identity will enhance academic self-efficacy. In addition, previous studies have focused mainly on Western European countries and the United States, but research in China, especially on preservice special education teachers, has been lacking. The training of preservice special education teachers in China puts more focus on theory than practice (Wang and Mu, 2014), whereas in the United States more attention is paid to practical experience (Cornelius and Nagro, 2014). The differences in training styles may affect the psychological state of preservice teachers. Because the practical experience gained by teachers can enhance their teaching efficacy, they can deal with the complicated nature of the teaching profession in the future with more confidence (Lombardo-Graves, 2014). Moreover, because the social status of special education teachers is lower than that of regular education teachers in China, this may lead to the low professional identity of preservice special education teachers and further negatively affect their ASE (Sun and Lei, 2016). Therefore, the purpose of this study is to explore the psychological state of Chinese preservice special education teachers; that is, the effects of students’ social support and perceived professional identity on ASE, particularly the possible role of professional identity in mediating between social support and ASE.

## Current Study

This study aims to contribute to the understanding of ASE and related sociodemographic factors, such as social support and professional identity, among Chinese preservice special education teachers. Specifically, its objective is to investigate the following hypotheses:

(1) H1a: Social support has a positive effect on professional identity.
H1b: Social support positively affects ASE.
H1c: Professional identity positively affects ASE.
(2) H2: Professional identity mediates the relationship between social support and ASE.

## Materials and Methods

### Participants

Chinese preservice special education teachers were selected from several universities, including South China Normal University, Lingnan Normal University, Guangdong Second Normal University, East China Normal University, and Southwest University, to answer questionnaires. This study used a convenient sampling method to distribute electronic questionnaires to these universities and then asked college students majoring in special education to fill in the questionnaires. A total of 322 questionnaires were collected and the recovery rate was 100%, of which 302 were valid and the effective rate was 93.7%. The participants included 58 males (19.2%) and 244 females (80.8%). The demographic characteristics of the participants are presented in Table 1.

**TABLE 1:** Demographic table of participants.

**Groups**	**Frequency**	**Percentage (%)**
**Gender**		
Male	58	19.2
Female	244	80.8
**Year level**		
Freshman	60	19.9
Sophomore	74	24.5
Junior	78	25.8
Senior	90	29.8
**Residence**		
Towns	123	40.7
Rural areas	179	59.3
**Type of school**		
Key colleges	121	40.1
General colleges	181	59.9

### Procedure

Cross-sectional surveys were conducted in Mainland China, and the participants were selected from various universities. The participants were provided with a detailed description of the study and the intended use of the results. The participants were invited to complete a set of questionnaires, including a sociodemographic information questionnaire, the Social Support Questionnaire for Preservice Special Education Teachers (Feng and He, 2014), the College Students’ Professional Identity Questionnaire (Qin, 2009), and the Academic Self-efficacy Scale (ASES) (Liang, 2000). The questionnaire data were kept confidential to protect the anonymity of the participants. All the student teachers voluntarily participated in the study and did not receive monetary compensation. All the participants provided their written informed consent to participate in this study, and the study was reviewed and approved by the Human Research Ethics Committee for Non-clinical Faculties (ethics committee from the South China Normal University) before it was conducted.

### Measures

#### Social Support Questionnaire for Preservice Special Education Teachers

The Social Support Questionnaire for Preservice Special Education Teachers was developed by He (2013). This questionnaire consists of 25 items and measures four dimensions: family support, teacher support, peer support, and environmental support. Family support refers to parents’ concern for and help with the emotional, material, information and other aspects of the daily life and professional learning of these student teachers (4 items, e.g., “My family cares about me and respects the professional choices I make”). Teacher support refers to the emotional, material and information care and help provided by teachers for these student teachers during their study (8 items, e.g., “I receive guidance from my teachers when I encounter learning problems”). Peer support refers to the concern for and help of friends and classmates for these student teachers’ emotional, material and information (7 items, e.g., “I receive considerable support and assistance from my friends and classmates during college”). Environmental support refers to the concern for and help given to these student teachers from the relevant policies and public opinions of the school and even the whole society (6 items, e.g., “The university provides a perfect environment for living and studying”). A Likert-type response scale from 1 (completely inconsistent) to 5 (completely consistent) was used to assess each item. He (2013) found that the Cronbach’s alpha of the entire questionnaire was 0.929, and that the questionnaire exhibited good structural validity. Confirmatory factor analysis (CFA) indicated that the fitness of the questionnaire was acceptable. Moreover, this scale demonstrated good reliability and validity in previous studies (Feng and He, 2014; Hu and Wang, 2017). In the current study, confirmatory factor analysis was performed with LISREL 8.7 software, and the results showed that NFI = 0.90, CFI = 0.92, IFI = 0.92, and RMSEA = 0.061. In addition, the Cronbach’s alpha coefficient of the entire scale was 0.92. The Cronbach’s alpha coefficients for family support, teacher support, peer support, and environmental support are 0.76, 0.87, 0.81, and 0.79, respectively. In terms of convergent validity, the composite reliability scores of all dimensions were higher than 0.70 (Malhotra and Dash, 2011). In discriminant validity, except for environmental support, all AVEs were higher than the squared inter-factor correlation (Marnburg and Luo, 2014). AVE of environmental support is 0.509, and the squared inter-factor correlation between environmental support and peer support is 0.582. This may be due to the fact that most of the environmental support college students perceive comes from peer support, because during college, environmental support also includes peer support without family members, which leads to their higher correlation coefficient.

#### College Students’ Professional Identity Questionnaire

The College Students’ Professional Identity Questionnaire was developed by Qin (2009), who referred to Brickson (2000). Brickson (2000) divided professional identity into four dimensions: cognitive, emotional, behavioral, and social. In a study of Chinese college students, Qin (2009) identified four dimensions: emotional, behavioral, appropriate, and cognitive identities. Emotional professional identity reflects the emotional preference for one’s major (8 items, e.g., “I have a positive emotional attachment to my major”). Behavioral professional identity refers to the level of professional behavior (6 items, e.g., “I take the initiative to participate in practical activities related to my major”). Appropriate professional identity reflects the matching degree between one’s major and oneself (4 items, e.g., “My personality matches my major”). Cognitive professional identity refers to the degree of understanding one’s major (5 items, e.g., “Overall, I know my major”). The questionnaire consists of 23 questions with answers ranging from 1 (completely inconsistent) to 5 (completely consistent). The higher the score, the stronger the sense of professional identity. Qin (2009) found that the consistency coefficient of the entire questionnaire was 0.944, and the consistency coefficient of each dimension was 0.574–0.766; thus, the questionnaire achieved good reliability. CFA showed that the structural validity of the questionnaire was acceptable. The results indicated that GFI = 0.904, CFI = 0.926, IFI = 0.926, and RMSEA = 0.057 (Qin, 2009). Moreover, this scale demonstrated good reliability and validity in previous studies (Zhang G. et al., 2016; Yang and Li, 2018). In the present study, the Cronbach’s alpha coefficient of the entire scale was 0.95. The Cronbach’s alpha coefficients for emotional, behavioral, appropriate, and cognitive identities are 0.78, 0.89, 0.86, and 0.84, respectively. Confirmatory factor analysis was performed with LISREL 8.7 software, and the results showed that NFI = 0.94, CFI = 0.96, IFI = 0.96, and RMSEA = 0.048. In terms of convergent validity, except for cognitive professional identity, the composite reliability scores of all dimensions were higher than 0.70 (Malhotra and Dash, 2011). The composite reliability of cognitive professional identity was 0.672. Relatively low compared to other dimensions, may be due to the abstract nature of individual item, such as “I know the outside evaluation of my major.” What’s the scope of this outside is not very clear. In discriminant validity, all AVEs were higher than the squared inter-factor correlation (Marnburg and Luo, 2014).

#### Academic Self-Efficacy Scale

Academic Self-Efficacy Scale was developed by Liang (2000). This scale consists of two dimensions: academic competence efficacy and academic behavior efficacy. Academic competence efficacy consists of 11 items and examines whether students are confident in mastering a subject matter and in achieving high scores. Academic behavior efficacy also consists of 11 items and examines students’ confidence in regulating their learning activities. A 6-point Likert scale ranging from 1 (completely inconsistent) to 6 (completely consistent) was used. The higher the score, the stronger the sense of ASE. This scale demonstrated good reliability and validity in previous studies (Liang, 2000; Shi et al., 2013; Xu et al., 2017). In the present study, the Cronbach’s alpha coefficients of the Academic Competence Efficacy Subscale and the Academic Behavior Efficacy Subscale were 0.92 and 0.70, respectively. The Cronbach’s alpha coefficient of the entire scale in this study was 0.90. Confirmatory factor analysis was performed with LISREL 8.7 software, and the results showed that NFI = 0.92, CFI = 0.94, IFI = 0.94, and RMSEA = 0.075. In terms of convergent validity, the composite reliability scores of all dimensions were higher than 0.70 (Malhotra and Dash, 2011). In discriminant validity, all AVEs were higher than the squared inter-factor correlation (Marnburg and Luo, 2014).

### Statistical Analyses

Statistical analyses were conducted using SPSS 22.0. First, we generated means, standard deviations (SDs), and a correlation matrix to explore the associations among the variables. Second, we performed hierarchical multiple regressions to determine the respective contributions of sociodemographic variables, social support, and professional identity to ASE. The demographic variables, including the residence, gender, type of school (key colleges and general colleges), and year level of student teachers were entered in Block 1 of the regression analyses. These demographic variables were set as dummy variables. Social support and professional identity were standardized and entered in Block 2. Lastly, we used SPSS 22 with process analysis (Hayes, 2013) to detect the mediation effect. For the model, the social support scores were specified as the independent variables whereas professional identity was specified as the mediator variable with gender, age, and type of schools as covariates. We used completely standardized indirect effects as a measure of magnitude (Preacher and Kelley, 2011) with large, medium, and small effects defined as 0.01, 0.09, and 0.25, respectively (Kenny, 2018). A *p*-value < 0.05 was considered statistically significant.

## Results

### Relationship Among Social Support, Professional Identity, and ASE

The means, SDs, and correlations of the study variables are presented in Table 2.

**TABLE 2:** Descriptive statistics and intercorrelations among variables.

	**1**	**2**	**3**	**4**	**5**	**6**	**7**
1. Gender	–						
2. Residence	0.024	–	–	–	–	–	–
3. Type of school	–0.073	0.037	–0.078				
4. Year level	0.046	–0.008	0.089				
5. Social support	0.089	–0.077	−0.175**	–0.005			
6. Professional identity	0.222**	0.008		0.035	0.684**		
7. ASE	0.128*	0.019	–0.061	0.141*	0.509**	0.670**	
Mean	1.81	1.59	1.60	2.66	3.777	3.705	3.378
SD	0.395	0.492	0.491	1.106	0.500	0.577	0.480

*N* = 302; **p < 0.01; *p < 0.05.

A significant positive correlation existed between social support and the total score of professional identity (*p* < 0.01); that is, the higher the social support, the higher the professional identity, and vice versa. Furthermore, a significant positive correlation was observed between social support and the total score of ASE (*p* < 0.01); that is, the higher the social support, the higher the ASE, and vice versa. Lastly, a significant positive correlation was also found between professional identity and the total score of ASE (*p* < 0.01); that is, the higher the professional identity, the higher the ASE. Therefore, a relationship exists among the social support, professional identity, and ASE of preservice special education teachers.

### Multiple Regression Analysis of ASE

Hierarchical multiple regression analyses were performed to examine the predictors of ASE. The results of the analyses are presented in Table 3. In Step 1 of the analysis, sociodemographic factors accounted for 3.7% (*R*^2^ = 0.037, *p* < 0.05) of the variance in ASE, whereas the gender and grade level of preservice special education teachers were statistically significant. The introduction of the social support and professional identity subscales in Step 2 accounted for 43.7% (*R*^2^ = 0.437, *p* < 0.001) of the variance in ASE. Together, Steps 1 and 2 accounted for 47.4% (*R*^2^ = 0.474, *p* < 0.001) of the variance in ASE, whereas grade and professional identity were statistically significant.

**TABLE 3:** Hierarchical multiple regression analysis results that predict ASE.

	**Step 1**		**Step 2**	
	**β**	***t***	**β**	***t***
Gender (1 = female, 2 = male)	0.118	2.066*	–0.019	–0.429
Residence (1 = urban areas, 2 = rural areas)	0.019	0.332	0.021	0.499
Type of school (1 = key colleges, 2 = general colleges)	–0.043	–0.756	0.064	1.486
Grade (1 = freshman, 2 = sophomore, 3 = junior, and 4 = senior)	0.132	2.316*	0.126	2.967**
Social support			0.103	1.758
Professional identity			0.611	10.166***
*R*^2^	0.037		0.474	
*F*	2.835*		44.251***	
Δ*R*^2^	0.024		0.437	
Δ*F*	2.835*		122.446***	

*p < 0.05, **p < 0.01, ***p < 0.001.

### Mediating Effect of Professional Identity on the Relationship Between Social Support and ASE

We tested for mediation by regressing the predictor variable, social support on ASE, while including the proposed mediator (professional identity). We first conducted these analyses by including the demographic variables identified earlier as control variables. Mediation analysis was conducted using 1000 bootstrap samples, which confirmed the significant direct effect of social support on ASE. As predicted, however, the result was rendered insignificant (*b* = 0.09, SE = 0.06, *t* = 1.64, *p* > 0.05) when the effect of professional identity was considered. This finding indicates that professional identity completely mediates the relationship between social support and ASE. The result of the Sobel (1982, 1986) test justified the mediating effect (*z* = 8.70, *p* < 0.001). As shown in Table 4, social support made a significant contribution to ASE (*b* = 0.49, SE = 0.05, *t* = 10.25, *p* < 0.001); that is, if the participants perceived more support, then their ASE would improve. Therefore, H1b was supported. Furthermore, social support made a significant contribution to professional identity (*b* = 0.79, SE = 0.05, *t* = 16.22, *p* < 0.001). When student teachers perceived more social support from others, they were more likely to report higher levels of professional identity in college. Then, the hypothesis H1a was confirmed. In turn, professional identity exerted a positive influence on ASE (*b* = 0.50, SE = 0.05, *t* = 10.33, *p* < 0.001). Accordingly, the hypothesis H1c was also confirmed. We corroborated that professional identity was a full mediator that explains the path from social support to ASE. In addition, we use G^∗^Power 3.1 software to perform power analysis on the whole model, and the results show that Cohen’s *f*^2^ = 0.89. According to Cohen (1988), the whole model has a strong effect.

**TABLE 4:** Mediating effect of professional identity on social support and ASE.

	***b***	**SE**	***t***	**LLCI**	**ULCI**
**ASE**					
Constant	0.00	0.02	0.00	–0.05	0.05
Social support	0.49	0.05	10.25***	0.40	0.58
	*R*^2^ = 0.26	*F* = 105.13***			
**Professional identity**					
Constant	0.00	0.02	0.00	–0.05	0.05
Social support	0.79	0.05	16.22***	0.69	0.88
	*R*^2^ = 0.47	*F* = 263.07***			
**ASE**					
Constant	0.00	0.02	0.00	–0.04	0.04
Social support	0.09	0.06	1.64	–0.02	0.20
Professional identity	0.50	0.05	10.33***	0.41	0.60
	*R*^2^ = 0.45	*F* = 106.89***			

***p < 0.001.

**FIGURE 1:**
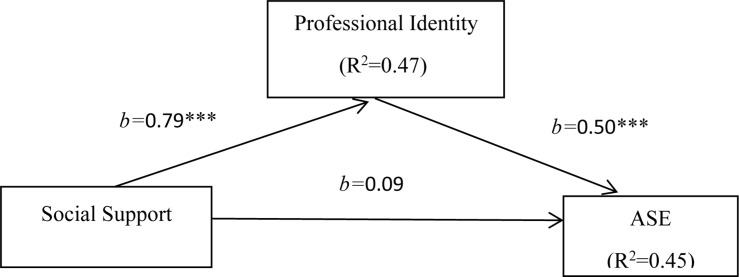
Mediation model. ^∗∗∗^*p* < 0.001.

This result confirmed the hypothesis H2 (i.e., professional identity plays a mediating role between social support and ASE). Therefore, we determined that professional identity mediated the relationship between social support and ASE. This model, i.e., Model 4 (Hayes, 2013) is illustrated in Figure 1.

## Discussion

Our study intended to broaden the current literature on ASE and related sociodemographic factors, such as social support and professional identity, among Chinese preservice special education teachers. In this study, ASE among preservice special education teachers is at a moderate level, which is consistent with the findings of Chen and Yang (2018). ASE is not ideal, which may be owing to a variety of factors. For example, the social support provided by universities in China may not be sufficient because there are not enough internships available (Guan et al., 2011). However, this study is not consistent with the findings of Gur (2012), which shows that Israel’s preservice special education teachers’ self-efficacy in learning is at the upper-middle level, while in this study it is close to the middle level. In addition, the students’ sense of professional identity is not very high, perhaps because many students do not opt for the special education major as their first choice when entering university (Sun and Lei, 2016). However, in China, the professional identity of regular teachers is in the upper middle level. Gong and Zhao (2016) studied 500 regular teachers in China, each item of professional identity in that study was measured according to a scale of 1–5, in which 1 was the lowest score,5 was the highest score and 3 scored at the intermediate level. The mean score of the participants was more than 4, exceeding the intermediate level.

Many people feel special education teachers have lower social status and lower income than regular teachers, which reduces their learning enthusiasm (Guan et al., 2011), and as such, their ASE is not high. Therefore, after these high school students enter university, teacher educators should provide support to them and strengthen their sense of professional identity to enable them to study hard, delve into the knowledge, and skills of special education, and gain competence in teaching special education in the future. Furthermore, this study found that as grade increased, their ASE also gradually increased. This is consistent with the previous research results of Gur (2012), whose study also found that with the increase in grade, the self-efficacy of preservice special education teachers improves. This may be because they have become familiar with the school’s teaching methods, mastered the learning strategies, and adapted to university life, thus enhancing their self-confidence and academic self-efficacy.

Similar to other studies, our analysis also showed a strong relationship between social support and professional identity (see Johnson and Ridley, 2004; Zhang and Wang, 2018) and between professional identity and ASE (see Chen and Yang, 2018). In accordance with Adler-Constantinescu et al. (2013), social support in this study is significantly correlated with self-efficacy. That is, the more social support an individual perceived, the higher the level of ASE. Social support is correlated significantly with – and can predict – professional identity. This finding is similar to the findings of Johnson and Ridley (2004). Providing preservice teachers with support, including initial instructional guidance for novice teachers, integrating them into the school’s culture, and communicating classroom programs with expert teachers can enhance their professional identity. Similarly, in a study of preservice teachers in China conducted by Zhao and Zhang (2017), mentor support in field teaching practice, as well as support from peers and friends, were found to be intuitively important for professional identity, because being helped and cared for by these important people allows students to identify gradually with the teaching profession. Furthermore, a significant correlation exists between professional identity and ASE. Preservice special education teachers that have a positive emotion toward professional identity will have a correct and positive attitude toward their own professional learning, can deal actively with the problems and difficulties in professional learning, and experience a higher sense of accomplishment and destination in professional practice, ultimately improving their own learning efficacy. Guo et al. (2017) also found that if nursing students have a negative personal identity, they consider work stress and poor environment to be important factors when thinking about their future employment. In this manner, if they believe that they cannot cope with their future career, they will have a low sense of career self-efficacy.

Our regression analyses support the hypothesis that professional identity completely mediates the relationship between social support and ASE. This phenomenon shows social support cannot predict ASE when the three variables are put together. These student teachers often have high ASE only after they identify with special education majors. The reason why professional identity is very important is that many people in China have a negative view of special education teachers, believing that they are poorer in ability and lower in social status than regular education teachers (Guan et al., 2011; Hu and Sang, 2013). If student teachers do not accept their major, even if they are provided with a lot of social support, they are not necessarily willing to study hard and their ASE will not be high.

The important mediating role of professional identity indicates that strengthening social support is insufficient and the effective educational strategies adopted by universities to improve the students’ sense of identity and belonging to their chosen majors should also be considered. By considering both social support and professional identity, students and teachers can feel more encouraged and confident, become more involved, and consequently enhance their ASE. Special education is an integral part of the education system and because of the special needs of students with disabilities, special education teachers require higher expertise and skills than regular teachers (Wang and Mu, 2014). Therefore, preservice teachers in China should be provided with social support in order to promote their professional commitment. Importance should be attached to their preservice practical exercises, adequate internships (field experiences) should be provided, and acceptance of children with disabilities also should be fostered. Greater recognition of special education teachers would lead to more successful entry into the workplace. The development of the professional identity and ASE of preservice special education teachers should also be retained in special education teaching. This is beneficial for them because good training and emotional stability will enable them to deal effectively with the complicated nature of special education teaching in the future.

## Limitations and Future Research

Three limitations of this study should be mentioned. The first limitation is the cross-sectional design of this study; the findings reflect associations, but not causal relationships among the variables (Maxwell and Cole, 2007; Mitchell and Maxwell, 2013). Longitudinal and experimental studies will provide additional insights into the relationships among these variables. The second limitation pertains to the data collected using self-reported scales. In future studies, the use of multiple evaluation methods may reduce the impact of subjectivity. The third limitation is that social support, professional identity and ASE are not further refined in this study; for example, social support can also be subdivided into peer support, family support and environmental support, and so on. Future studies may further analyze these variables and explore the relationship between the various fractal dimensions. Future experimental studies in which variables can be manipulated will enable us to draw stronger conclusions regarding causal relationships between social support and ASE.

## Conclusion

The ASE level of preservice special education teachers is not ideal. A significant positive correlation exists among the ASE, social support, and professional identity of undergraduates majoring in special education. Professional identity plays a mediating role between social support and ASE. Therefore, professional identity and perceived social support are crucial for improving an individual’s sense of ASE. In particular, social support positively influences ASE via professional identity.

## Data Availability Statement

The datasets generated for this study will not be made publicly available as permission was not granted in the consent. Permission to access the data can be made by contacting the corresponding author (zhongjingxun@sina.com).

## Author Contributions

XC and JZ provided the idea, designed this study, and wrote the manuscript. MiL and MaL contributed to data analysis and data collection. MiL revised this manuscript.

## Conflict of Interest

The authors declare that the research was conducted in the absence of any commercial or financial relationships that could be construed as a potential conflict of interest.
